# Comparing Cardiovascular Events Across Home Dialysis Modalities: Adjusting the Unadjustable?

**DOI:** 10.34067/KID.0000000000000397

**Published:** 2024-04-25

**Authors:** Annie-Claire Nadeau-Fredette, Jenny I. Shen, Jeffrey Perl

**Affiliations:** 1 Department of Medicine, Université de Montréal, Montreal, Quebec, Canada; 2 Hôpital Maisonneuve-Rosemont Research Center, Montreal, Quebec, Canada; 3 Division of Nephrology, Hypertension, and Transplantation, The Lundquist Institute at Harbor-UCLA Medical Center, Torrance, California; 4 Division of Nephrology, St. Michael's Hospital, Unity Health, University of Toronto, Toronto, Ontario, Canada

**Keywords:** chronic dialysis, clinical epidemiology, hemodialysis, peritoneal dialysis

Cardiovascular disease is the leading cause of mortality in individuals receiving dialysis with rates of cardiovascular-related mortality nearly 20 times higher compared with those in the general population.^1^ Reasons underpinning this increased risk may include a higher prevalence of predialysis cardiovascular disease and associated risk factors (diabetes, hypertension, obesity, *etc.*) in patients with advanced CKD and the added burden of unique potential uremia-associated cardiovascular risk factors (*i.e*., hyperphosphatemia) once reaching advanced kidney failure.^2^

Previous studies have shown that home hemodialysis (HHD), especially if performed as intensive (long hours and/or frequent) hemodialysis, is associated with cardiovascular benefits when compared with conventional facility hemodialysis. Patients treated with HHD have been shown to have improvements in left ventricular geometry, serum phosphorus, and BP control.^3-5^

While the increased burden of cardiovascular disease and related adverse events are well known among patients with kidney failure, very few studies have specifically evaluated its comparative association across home dialysis modalities. At this time, the literature comparing peritoneal dialysis (PD) and HHD has concentrated on mortality, modality transfer, and, to a lesser extent, hospitalizations, showing mostly similar or more favorable outcomes with HHD compared with PD.^6–8^ Albeit, many of these adverse events reported in prior studies are likely cardiovascular in nature.

In the 5.2 of *Kidney360*, Shah and colleagues compared cardiovascular events in a large cohort of individuals treated with either PD (*n*=63,931) or HHD (*n*=4714) within 6 months of RRT initiation in the United States. This large study not only assessed overall cardiovascular-related hospitalizations (as previously reported in smaller cohorts) but also informed on individual cardiovascular events requiring admission, such as heart failure, myocardial infarction, and stroke.

Shah and colleagues showed higher rates of crude cardiovascular events in patients receiving HHD compared with PD (127.8 per thousand person-years in HHD versus 93.3 per thousand person-years in PD). After adjustment for a variety of available demographic and comorbid conditions, a reverse association was seen with an 8% lower risk of cardiovascular events in patients treated with HHD versus PD. Exploring individual cardiovascular admission events, the adjusted risk of hospitalization for stroke and acute coronary syndromes was lower in patients on HHD while the risk of heart failure was similar across the two home dialysis modalities. All-cause and cardiovascular-related mortality also presented a reverse association with higher rates in patients treated with HHD in crude analyses, but lower risk with HHD in adjusted regressions.

We praise Shah and colleagues for their meticulous examination of cardiovascular outcomes in patients receiving home dialysis in this large expansive dataset. However, the study findings need to be interpreted in the context of the study design and characteristics of the population assessed. First, we take note that characteristics of patients in the HHD cohort appear somewhat different than previously described HHD populations. In this study, 20% patients on HHD were 80 years and older.^7,8^ Overall, this translated into older individuals receiving HHD compared with PD (age 68±13 versus 63±15 years); having more cardiovascular disease (50.3% versus 37.5%); and having a higher proportion of diabetes (56.5% versus 51.9%), greater proportion of poor functional status (29.1% versus 5.7%), and lower serum albumin (3.3 versus 3.5 g/dl). This is in direct contrast to a previous study published by Weinhandl and colleagues in the United States where patients treated with HHD were significantly younger than their PD counterparts (53.6±14.7 versus 56.2±19 years).^7^

As detailed by the authors, the older age in the HHD cohort in this study compared with prior studies is likely related to inclusion of a significant proportion of patients receiving hemodialysis in skilled nursing facilities. These elderly and frail institutionalized patients are not on self-care and traditionally have higher rates of short-term adverse events, including cardiovascular events, hospitalization, and deaths. In fact, in the study by Shah *et al.*,^9^ residence in a nursing home was associated with a 1.41 times increased risk of cardiovascular events compared with non–nursing home residents. Incorporating this skilled nursing facility population to the typical HHD cohort may have resulted in the observed higher crude rate of cardiovascular events, hospitalizations, and deaths observed in the HHD compared with PD cohort. Even adjusting for available covariates may still leave significant residual confounding. Lack of data on noninstitutionalized assisted home dialysis may also be involved and could not be accounted for because these data are not captured in the United States Renal Data System. Moreover, we do not see the proportion of nursing home residents by dialysis modality listed in the study. The authors did attempt to remove 2631 individuals receiving care in a facility, with >90% of patients residing in a skilled nursing facility in the analysis. However, a sensitivity analysis using nursing home facility as a time-varying covariate and censoring at that time would have been valuable in comparing outcomes. In addition, patients on private insurance were not included in this study, which may have introduced a selection bias because they may be overrepresented among younger and working individuals.

Another cohort-related restriction involved the exclusion of patients with <3 months of home dialysis treatment, leading to nearly 20% of the cohort being excluded. This exclusion is important because adverse events are more frequent during the first few weeks after dialysis start (or modality change). These excluded patients likely experienced an important number of cardiovascular events.^10,11^

The second reason why exclusion of early outcomes is important is related to differences in the typical timing of home dialysis initiation between patients on PD versus HHD. Most patients initiate PD as their first RRT (or shortly after a brief period of facility-based hemodialysis) while HHD is usually started after some time on facility hemodialysis (including training time). Early dialysis-related adverse events may have been more frequently attributed to the PD cohort than HHD (because patients were not on HHD at that time). This could also have contributed to the poorer cardiovascular outcomes found in PD compared with HHD.

The third reason why exclusion of early events may affect comparisons between PD and hemodialysis relates to the possibility that dialysis vintage may be an important modifier in the relationship between home dialysis modality and outcomes. Indeed, in a previous study by Weinhandl and colleagues, daily HHD was associated with a lower risk of hospitalization and mortality compared with PD; however, when the cohort was restricted to incident patients (with study inclusion within 6 months of RRT initiation), outcomes were similar.^7^ In this regard, dialysis vintage may be a proxy for residual kidney function (RKF), which may also be involved in the observed adverse event differences by home dialysis modality over time. Indeed, RKF is well known for its association with improved outcomes in PD possibility mediated by enhanced sodium and fluid removal, improved left ventricular remodeling, and improved solute removal.^12-14^ PD typically is associated with greater preservation of RKF and urine volume compared with facility-based hemodialysis, which is considered one of the benefits of PD as the first RRT modality. By contrast, intensive HHD has been shown to precipitate urine volume decline.^15^ The benefits of home and intensive HHD may be best realized in longer vintage patients who have lost RKF and rely exclusively on solute and fluid removal by dialysis. An analysis in the study by Shah *et al.*^9^ stratified by dialysis vintage would have provided important insights.

In the study by Shah *et al.*,^9^ HHD dose and prescription patterns (weekly number and duration of HHD sessions) were unavailable. Detailed HHD prescription data would have provided critical information knowing that both more frequent sessions (mainly related to prevention of the 2-day dialysis gap), which have been associated with an increased risk of death, and longer sessions have been associated with cardiovascular benefits.^16^ Hence, future studies should aim for more granular HHD prescription-related data, which could inform optimal practices for patients undergoing home dialysis in future.

It is clear that both PD and HHD have a relatively high risk of cardiovascular events. In this study, after adjustment for potential confounding, dialysis modality had a very small association with heightened cardiovascular risk, but other modifiable factors such as neighborhood poverty and smoking had much stronger associations. Strategies that address disparities in care are needed independent of dialysis modality to prevent cardiovascular events in this high-risk population. As we try to individualize dialysis treatment to each patient's goals and preferences, this small difference seen across home dialysis modalities in the present study should not be the primary driver of home dialysis modality choices. Further investigation to compare outcomes across home dialysis modalities using observational data will continue to attempt to adjust for the unadjustable differences in the individuals who choose these varying treatment options. Instead, we should shift the focus to a patient-centered, integrated home dialysis approach. Such an approach views PD and HHD as complementary therapies that may be considered equally in the dialysis modality decision-making process in the vast number of patients. Both PD and HHD should perhaps even be used successively to leverage the potential benefits of both therapies and allow patients to continue to enjoy the enhanced autonomy and quality of life offered by receiving dialysis at home.
